# Micro-thin hydrogel coating integrated in 3D printing for spatiotemporal delivery of bioactive small molecules

**DOI:** 10.1088/1758-5090/ad89fe

**Published:** 2024-11-11

**Authors:** Md Sarker, Soomin Park, Vivek Kumar, Chang H Lee

**Affiliations:** 1 Biomedical Engineering, University of Maryland Eastern Shore, 30665 Student Services Center, Princess Anne, MD 21853, United States of America; 2 Center for Dental and Craniofacial Research, College of Dental Medicine, Columbia University Medical Center, 630 W. 168th Street, VC12-210, New York, NY 10032, United States of America; 3 Department of Bio-Medical Engineering, New Jersey Institute of Technology, 138 Warren St., Room 316, Newark, NJ 07102, United States of America

**Keywords:** tissue engineering, spatiotemporal delivery, bioactive scaffold, bioprinting

## Abstract

Three-dimensional (3D) printing incorporated with controlled delivery is an effective tool for complex tissue regeneration. Here, we explored a new strategy for spatiotemporal delivery of bioactive cues by establishing a precise-controlled micro-thin coating of hydrogel carriers on 3D-printed scaffolds. We optimized the printing parameters for three hydrogel carriers, fibrin cross-linked with genipin, methacrylate hyaluronic acid, and multidomain peptides, resulting in homogenous micro-coating on desired locations in 3D printed polycaprolactone microfibers at each layer. Using the optimized multi-head printing technique, we successfully established spatial-controlled micro-thin coating of hydrogel layers containing profibrogenic small molecules (SMs), Oxotremorine M and PPBP maleate, and a chondrogenic cue, Kartogenin. The delivered SMs showed sustained releases up to 28 d and guided regional differentiation of mesenchymal stem cells, thus leading to fibrous and cartilaginous tissue matrix formation at designated scaffold regions *in vitro* and *in vivo*. Our micro-coating of hydrogel carriers may serve as an efficient approach to achieve spatiotemporal delivery of various bioactive cues through 3D printed scaffolds for engineering complex tissues.

## 1. Introduction

Three-dimensional (3D) printed scaffolds have been extensively explored in tissue engineering and regenerative medicine to reconstruct the complex microstructures of native tissues [1–4]. Recently, 3D-printed scaffolds have evolved to incorporate a controlled delivery of various bioactive cues for facilitating tissue regeneration [2–6]. Bioactive cues such as growth factors (GFs), small molecules (SMs), and gene modifiers have been widely used along with 3D scaffold to guide cell differentiation, and spatiotemporally controlled delivery of those agents has been frequently considered to engineer heterogeneous, complex tissues [4–9].

Given its unique advantages in the precise positioning of biomaterials, 3D printing is considered effective for spatially controlled delivery of GFs and other bioactive cues [6]. As a simple and straightforward approach, GFs can be 3D-printed as functionalized or physically entrapped in the printing materials [10, 11]. However, this approach is limitedly applicable to hydrogel-based biomaterials that exhibit soft mechanical properties and are not suitable for mechanically demanding target tissues [12]. To address the limitation, a hybrid approach can be applied to deliver GFs by infusing hydrogels into the pores of 3D-printed solid scaffolds [13]. For example, chondrogenic parathyroid hormone-loaded hydrogel was applied in between 3D-printed polycaprolactone (PCL) fibers to construct scaffolds delivering the factors [14]. Another study infused agarose and gelatin hydrogel loaded with bone morphogenetic proteins (BMP)-2 into hollow cylindrical PCL/poly(lactic-co-glycolic acid) (PLGA) scaffolds for bone segment defect healing [15]. However, the hydrogel infusion into the pores can suffer from a limited spatial control of delivery, potential interference with the desired effect of the scaffold’s micro-pattern on cell activities, and technical challenges to achieve a prolonged release due to quick degradation rate of hydrogels [6].

To overcome these limitations, we have recently devised a micro-precise spatiotemporal delivery system integrated into the 3D-printing process [3]. PLGAs microspheres (*µ*S) encapsulated with various GFs were embedded in 3D-printed PCL fibers [3]. This US-patented approach [16] enabled the delivery of sustain-releasing GFs in different regions in 3D printed scaffolds, consequently leading to the regeneration of integrated multiphase tissues from a single type of stem/progenitor cells [3, 17, 18]. Despite the promising outcomes in the regeneration of complex tissues, including tendon-to-bone interface, knee meniscus, and temporomandibular joint discs, we realized several limitations of our previous approach. First, degradation byproducts of PLGA may lead to acidic environment, potentially interfering with tissue healing [19]. Second, a relatively fast degradation of PLGA *µ*S may accelerate the degradation of PCL, potentially resulting in an unpredictable *in vivo* degradation of scaffold structure [1, 3, 5]. Lastly, PLGA *µ*S has been widely used for controlled delivery of GFs, but its efficacy in micro-encapsulation and prolonged release of SMs has not been well-established [20].

In this study, we explored a new strategy for spatiotemporal delivery of SMs by establishing precisely controlled micro-thin coating of hydrogel carriers on desired locations of PCL layers as an integrated 3D printing process using a multiple head extrusion system (MHES). We optimized 3D printing parameters and material compositions to establish spatially controlled, homogenous micro-thin layers of selected hydrogel carriers. Profibrogenic SMs, Oxotremorine M (Oxo-M) and PPBP maleate (4-PPBP), and chondrogenic SM, Kartogenin (KGN), were successfully delivered as integrated at desired locations in 3D PCL scaffolds. The spatially delivered SMs showed prolonged release, leading to regional differentiation of mesenchymal stem cells (MSCs) and multiphase fibrocartilaginous tissue formation *in vitro* and *in vivo*. We selected Oxo-M and 4-PPBP for their robust effect on inducing fibrogenic differentiation [21] and KGN for its well-established function in chondrogenesis [22] to test a formation of multiphase fibrocartilaginous tissue formation.

## 2. Methods

### 2.1. Establishing homogenous micro-thin coating of hydrogel carriers on 3D printed PCL microfibers

**Figure 1.**
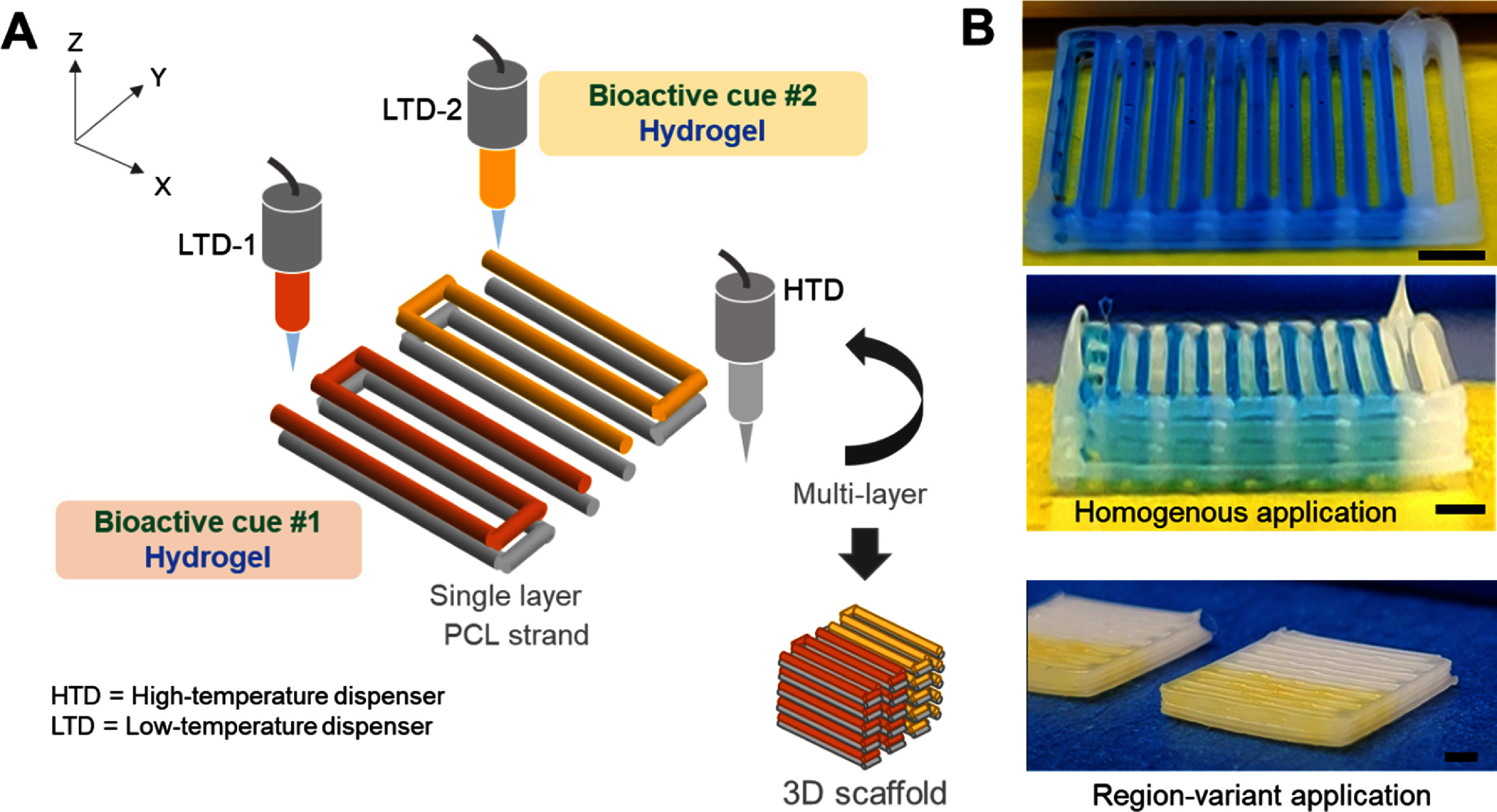
Micro-thin hydrogel coating approach for spatiotemporal delivery of multiple bioactive cues. Schematics of spatially controlled hydrogel coating method on PCL scaffold with a multiple head extrusion system (MHES) (A). Representative 3D-printed PCL scaffolds with micro-thin hydrogel coating for homogenous and regional-variant application (B). Colored dyes were mixed with HAMA hydrogel for visualization. Scale = 2 mm.

We selected several coating materials widely used as delivery vehicles, including fibrin-crosslinked with genipin (FibGen) [23, 24], methacrylate hyaluronic acid (HAMA) [25, 26], and multidomain peptides (MDPs) [27]. Hydrogel coating materials were printed on top of 3D-printed PCL (PURASORB^®^ PC 12, Corbion, Lenexa, KS) filaments using the MHES integrated 3D Bioplotter™ (EnvisionTec, Germany) per our established methods (120 °C; 6–8 PSI; 2–3 mm s^−1^) [28, 29]. As a testing template for the printing optimization, 400 *µ*m PCL filaments were printed in a monolayer using a high-temperature dispenser (HTD), followed by extruding hydrogels following matched printing coordination using a low-temperature dispensing unit (LTD) as outlined in figure 1(A). This approach enables exchanges of multiple LTDs containing different types of hydrogel and bioactive cues in a single printing process, allowing delivery of multiple factors at different locations. As demonstrated in figure 1(B), this method can form 3D constructs made of layers with a homogenous coating of hydrogel carriers, either with one type of bioactive cue or spatially delivered multiple factors.

For FibGen, fibrinogen was first printed on top of PCL filaments, followed by cross-linking with thrombin (200 U ml^−1^) and genipin (2.5 mg ml^−1^). The extruded HAMA was photo-crosslinked in the presence of lithium phenyl-2,4,6-trimethylbenzoylphosphinate (LAP, 0.4% w/v) and UV light (10 min exposure; 50 mW cm^−2^). MDP was self-assembled into beta-sheets, thus requiring additional cross-linking methods as described in our previous studies [30, 31]. For all hydrogel polymers, the inner diameter of the dispensing nozzle and the extrusion pressure were set to 250 *µ*m and 0.2 bar, respectively. Hydrogel printing was performed at room temperature (RT), whereas PCL was printed at 120 °C. As being dispensed through the HTD, PCL fibers are promptly cooled down to RT, thus resulting in no harmful effect on the bioactivity of SMs loaded in hydrogel given 2–3 min interval between PCL printing and micro-coating. To achieve the desired coating thickness and surface coverage, a range of hydrogel concentrations and printing parameters, including printing speeds and feeding rate, were tested (supplementary figure 1(A)). We performed initial printability tests for 0.5%–2% MDP with 7–28 mm s^−1^ printing speeds, 16%–20% HAMA with 0.5–6 mm s^−1^ printing speeds, and 18%–22% fibrinogen with 7–12 mm s^−1^ printing speeds (supplementary figure 1(A)). After excluding the parameters resulting in over-printing (supplementary figure 1(B)) and under-printing (supplementary figure 1(B)), we further tested three printing speeds per each selected concentration of hydrogel.

### 2.2. Evaluation of the micro-coated hydrogel layers

For quantitative measurements of the thickness and distribution of the micro-coating layers, confocal microscopy was used with fluorescein isothiocyanate (FITC)-labeled hydrogels. After imaging using a high-resolution confocal microscope (Leica TSC SP8), the thicknesses of hydrogel over PCL filament were quantified using Imaris software (Oxford Instruments, Abingdon, UK). Then, the contours of thickness distribution were graphed using Origin 2019 (OriginLab Corp., Northampton, MA). Based on the thickness contours, the printing speed exhibiting the most homogeneous hydrogel coating was selected for each hydrogel material that was then used for mechanical tests and release kinetics, as described below. To measure the bonding strength of hydrogel coating, we performed a lap shear test by applying a micro-coating of selected hydrogel layer between 3D printed, monolayered PCL membrane, followed by applying constant displace at 1 mm s^−1^ using a UniVert testing system (CellScale, Waterloo, ON).

### 2.3. SMs delivery and release kinetics

Oxo-M (200 mM), 4-PPBP (25 mM), or KGN (78.8 mM) was delivered via micro-coating of hydrogel prepared at the optimal composition determined above [21, 32]. The doses of SMs were selected as pre-optimized from our previous studies in delivering via hydrogel for induction of fibrogenic and chondrogenic differentiation of MSCs [21, 22, 32]. The *in vitro* release profiles of SMs were determined by immersing the hydrogel-coated scaffold in PBS for four weeks, followed by measuring concentrations of released SMs in collected media at selected time points using UV spectrophotometers per our previous methods [21, 32]. The cumulative release kinetics were calculated as a percentage of initial loading amount.

### 2.4. Formation of multiphase fibrocartilaginous tissue matrix

To assess tissue formation capacity in target tissue types, PCL scaffolds (10 mm × 10 mm × 2 mm; length × width × height) were prepared with 600 *µ*m pores by 3D printing, as either FibGen or HAMA micro-coating was applied to each layer using the MHES printing. Oxo-M (200 mM) and 4-PPBP (25 mM), were embedded in the hydrogel coating in half of each scaffold for fibrogenesis, whereas KGN (78.8 mM) was delivered in the other half for chondrogenesis. MDP hydrogel was not tested for tissue formation, given its low loading yield and release properties in comparison with HAMA and FibGen. The scaffolds were sterilized using ETO gas and then seeded with human bone marrow-derived MSCs (AllCells, Alameda, CA) by applying cell-loaded type I collagen gel (P2-3, 8 × 10^6^ cells ml^−1^) into the pores. The MSC-seeded scaffolds were then cultured for 4 weeks in 1:1 mixture of fibrogenic and chondrogenic induction media per our previous works [18, 24, 29]. In addition, the MSC-seeded scaffolds were implanted subcutaneously in immunodeficient mice for 4 weeks. Scaffolds micro-coated with HAMA or FibGen without SMs were tested as a control. After harvesting, tissue formation was evaluated using quantitative reverse transcription polymerase chain reaction (qRT-PCR) for mRNA expressions of fibrous and chondrogenic markers, including collagen type I & III (COL-I & III), scleraxis (SCX), Mohawk (MKX), SOX-9, and aggrecan (ACAN). Total collagen and glycosaminoglycans (GAGs) contents were measured using commercially available assay kits per our previous works [3, 17, 24, 29, 33]. Histological analysis was performed with H&E, Picrosirius Red (PR) with polarized microscopy, and Alcian Blue (AB). Quantitative collagen fiber orientation was measured using FiberFit™ software with PR-stained polarized images per prior works [34]. Immunofluorescence was performed for COL-I and ACAN per our prior works [3, 17, 24, 29, 35].

### 2.5. Statistical analysis

All quantitative data from all groups were analyzed by one-way ANOVA with Bonferroni multiple comparison tests at α level of 0.05 after confirmation of normal data distribution using PRISM ver. 11 (GraphPad Software, San Diego, CA).

## 3. Results

### 3.1. Optimized printing parameters for spatial delivery via hydrogel micro-coating

**Figure 2.**
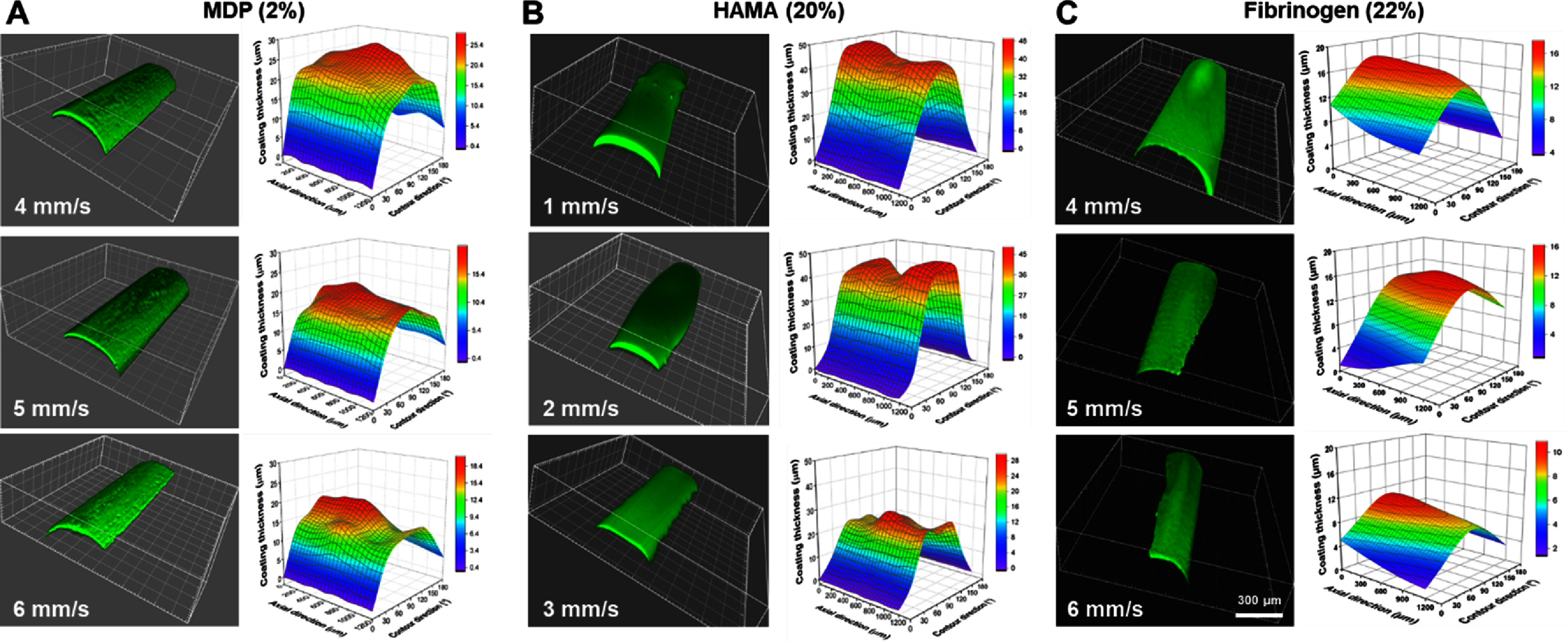
Confocal image analysis for micro-thin hydrogel coating layers with different printing speeds. Pre-determined concentrations of MDP (2%) (A), HAMA (20%) (B), and Fibrinogen (22%) (C) were tested at selected printing speeds. The FTIC-dyed hydrogel was used for visualization with confocal microscopy, and measured thicknesses along with axial direction and semi-circular locations were used to generate the thickness contour graphs. MDP (2%) with 1 mm s^−1^, HAMA (20%) with 1 mm s^−1^, and Fibrinogen (22%) with 4 mm s^−1^ showed the most homogenous coating layers.

The initial printability tests determined the ranges of printable material concentrations and printing speeds for each hydrogel material (supplementary figure 1(A)). In general, low material concentrations with slow printing speeds ended up with over-printing (supplementary figure 1(B)), and high material concentrations with higher printing speeds led to under-printing (supplementary figure (C)). From the initial testing, we identified the most effective printing speed with hydrogel concentrations for each biomaterial: 2% MDP with 4-6 mm s^−1^, 20% HAMA with 1–3 mm s^−1^, and 22% fibrinogen with 4–6 mm s^−1^ (supplementary figure 1(A)). Confocal microscopic analysis with the selected formulations showed that 2% MDP with 1 mm s^−1^, 20% HAMA with 1 mm s^−1^, and 22% fibrinogen with 4 mm s^−1^ result in the most homogeneous and consistent micro-thin coating of hydrogel layers on PCL filaments (figure 2). Quantitative analysis of the coating thicknesses performed along with axial direction and contour direction (in angle) (supplementary figure 2(A)) consistently demonstrated that 2% MDP with 5 mm s^−1^, 20% HAMA with 1 mm s^−1^, and 22% fibrinogen with 4 mm s^−1^ exhibit the most reliable and homogeneous coating (figure 2). The selected concentrations and printing speeds for MDP, HAMA, and FibGen resulted in coating thicknesses of ∼26 *µ*m, ∼48 *µ*m, and ∼18 *µ*m, respectively (figure 2 and supplementary figure 2(B)). Quantitatively, the standard deviation of coating thickness at 16 randomly selected points was significantly lower in 2% MDP with 5 mm s^−1^, 20% HAMA with 1 mm s^−1^, and 22% fibrinogen with 4 mm s^−1^ than the other groups (supplementary figure 2(C)). These data were confirmed by *n* > 30 repetitions using each set of printing parameters, which result in homogenous outcome with less than 5% inter-sample variance.

### 3.2. Lap shear properties and release kinetics

**Figure 3.**
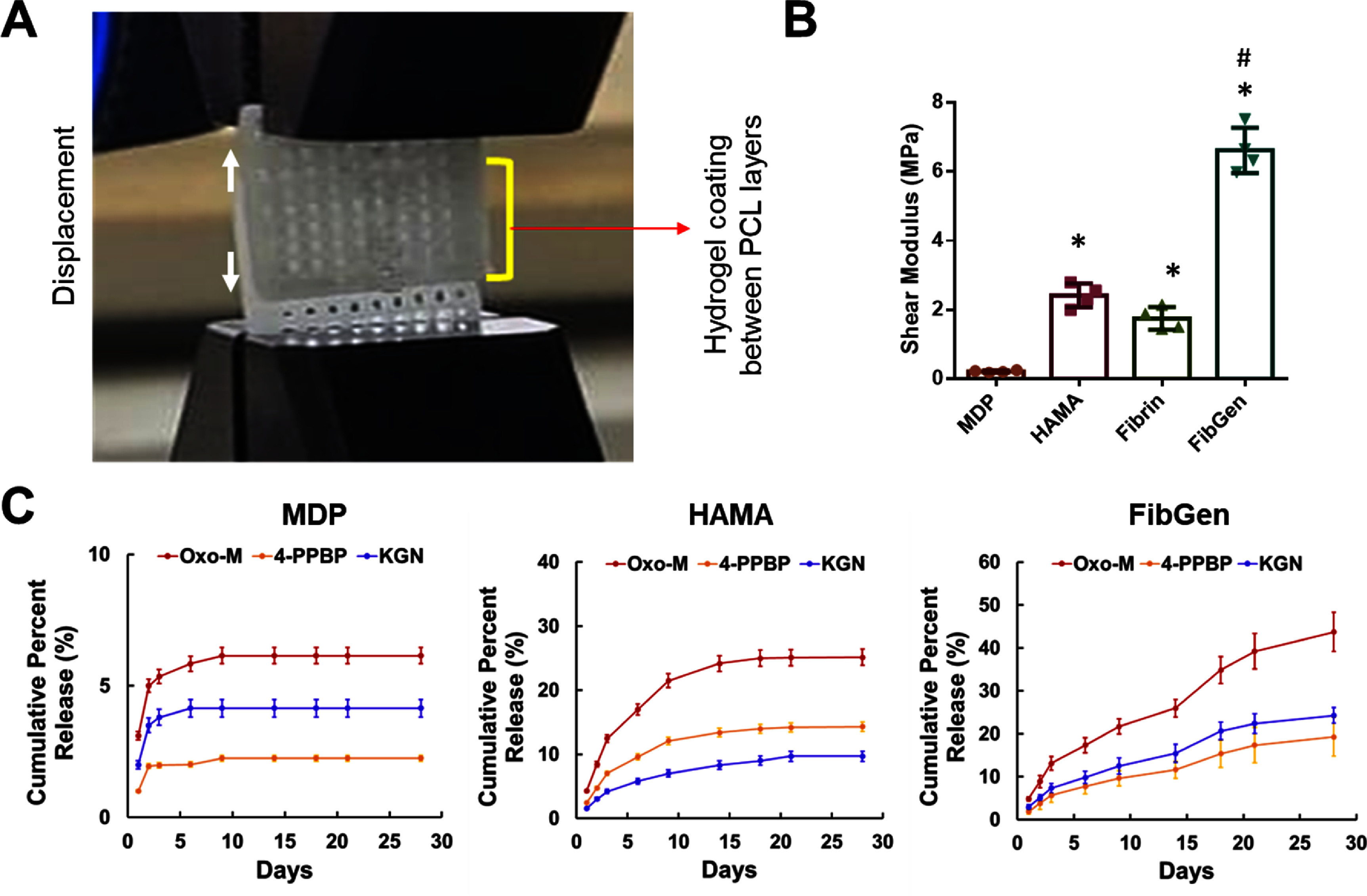
Evaluation of lap shear strength of hydrogel coating and release kinetics of delivered SMs. Lap shear tests were performed with hydrogel coating applied between two 3D-printed PCL membranes (A), resulting in shear moduli significantly higher with HAMA, fibrin, and FibGen than MDP (B) (*n* = 5 per group; *:*p* < 0.001 compared to MDP; #:*p* < 0.001 compared to all the other groups). *In vitro* release kinetics of Oxo-M, 4-PPBP, and KGN showed sustained release up to 10–28 d (C) (*n* = 5 per group & time point).

Lap shear test performed with hydrogel coating between two layers of 3D-printed PCL sheets (figure 3(A)) demonstrated that shear moduli of HAMA, Fibrin (no genipin cross-linking), and FibGen were significantly higher than MDP, with FibGen showing >3 fold higher shear modulus than HAMA and Fibrin (figure 3(B)) (*n* = 4 per group; *p* < 0.001). *In vitro* release tests showed that MDP released up to 2%–6% of initially loaded SMs with the release plateau reached around 7 d for all three SMs tested (figure 3(C)). HAMA showed sustained release of SMs up to 28 d, with noticeable plateau at 14, 18, and 21 d for Oxo-M, 4-PPBP, and KGN, respectively (figure 3(C)). FibGen showed sustained release without reaching a plateau by 28 d (figure 3(C)).

### 3.3. Multiphase fibrocartilaginous tissue formation

**Figure 4.**
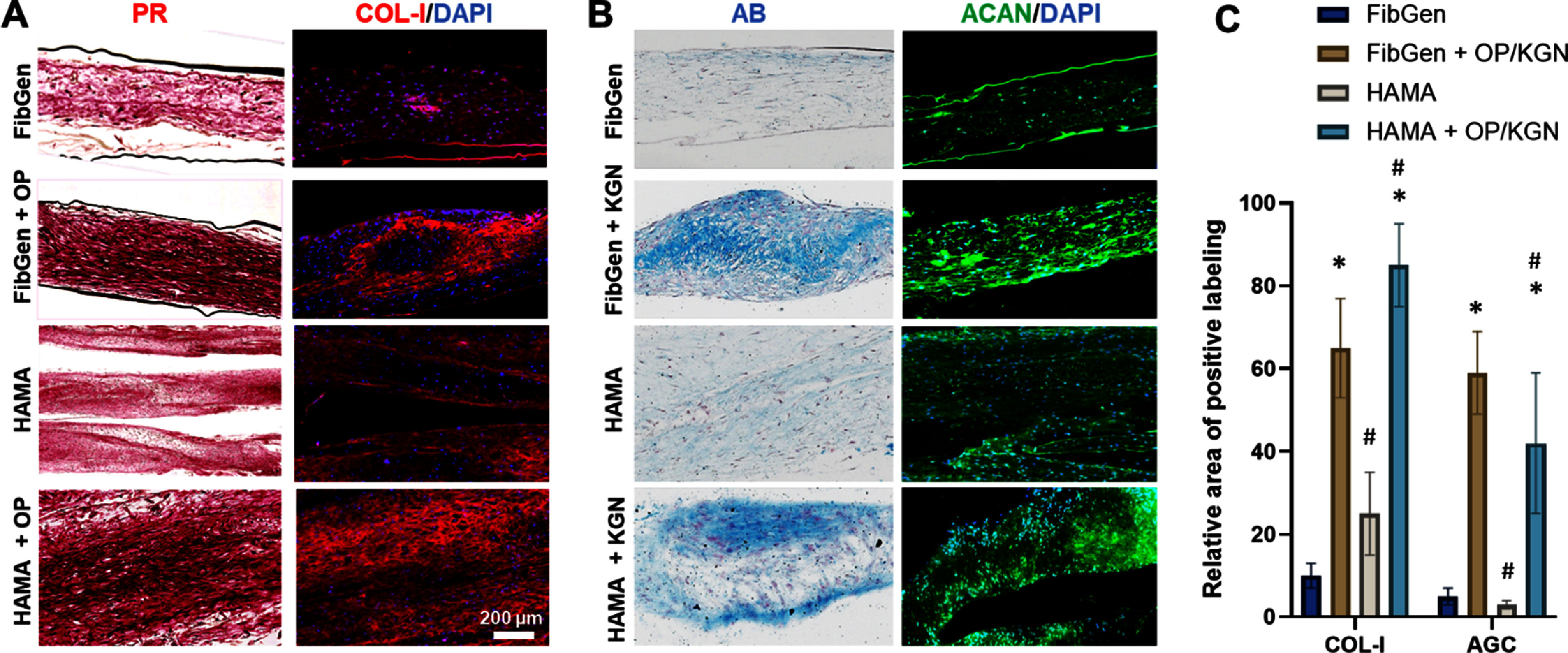
*In vitro* formation of multiphase fibrocartilaginous tissue by 4 weeks culture of PCL scaffolds spatially coated with Oxo-M & 4-PPBP (OP) and HAMA. Picrosirius Red (PR) staining and immunofluorescence of COL-I show dense collagenous tissue matrix formed at regions coated with FibGen + OP and HAMA + OP, as compared to control with hydrogel coating alone (A). Similarly, Alcian Blue (AB) staining and immunofluorescence of aggrecan (ACAN) showed cartilaginous matrix formation at the regions delivered with KGN (B). Quantitatively, COL-I+ area and AGC+ area are significantly higher in OP and KGN delivery, respectively, in both types of hydrogels (C) (*n* = 10 per group; *:*p* < 0.001 compared to hydrogel alone control; #:*p* < 0.001 compared to the other hydrogel).

**Figure 5.**
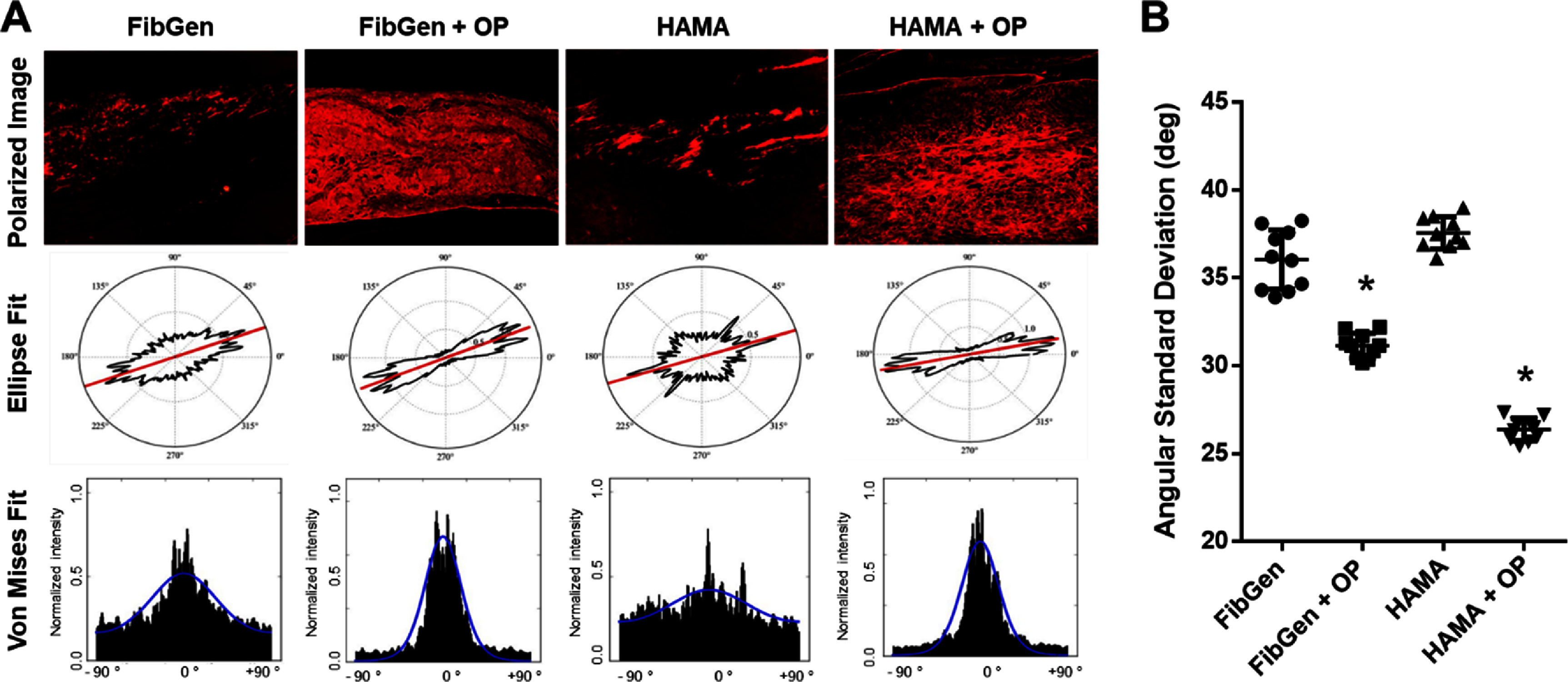
FiberFit™ analysis for collagen fiber organization. Polarized PR-stained images were analyzed using FiberFit™, resulting in Ellipse Fit and Von Mises Fit visualizing the distribution of fiber orientation angles (A). Quantitatively, angular standard deviations were significantly lower in the regions coated with FibGen + OP and HAMA + OP as than hydrogel alone controls (B) (*:*p* < 0.001 compared to control without OP; *n* = 10 per group).

Histology of *in vitro* tissue constructs showed dense collagenous tissue matrix in scaffolds delivery with Oxo-M and 4-PPBP via FibGen or HAMA as compared to control without SMs (figure 4(A)). Consistently, immunofluorescence showed tissue matrix with abundant COL-I in scaffolds delivered with Oxo-M and 4-PPBP via FibGen or HAMA, as compared to control (figure 4(A)). Similarly, AB staining showed cartilaginous matrix formation in scaffold with FibGen + KGN or HAMA + KGN in comparison with control without SMs (figure 4(B)). Consistently, immunofluorescence showed abundant ACAN expression in the scaffolds delivered with KGN via FibGen or HAMA (figure 4(B)). Quantitatively, relative areas positive for COL-I and AGC measured by ImageJ were significantly higher in the SM-delivery group than hydrogel alone groups (figure 4(C)). FiberFit™ analysis demonstrated that dense fibrous tissue formed in the scaffold regions delivered with Oxo-M and 4-PPBP showed an enhanced fiber organization and alignment in comparison to control (figure 5(A)). Quantitatively, the fiber angular standard deviations were significantly smaller with Oxo-M and 4-PPBP delivery than control without SMs (figure 5(B)) (*p* < 0.001; *n* = 10 per group).

### 3.4. mRNA expressions and matrix assays

**Figure 6.**
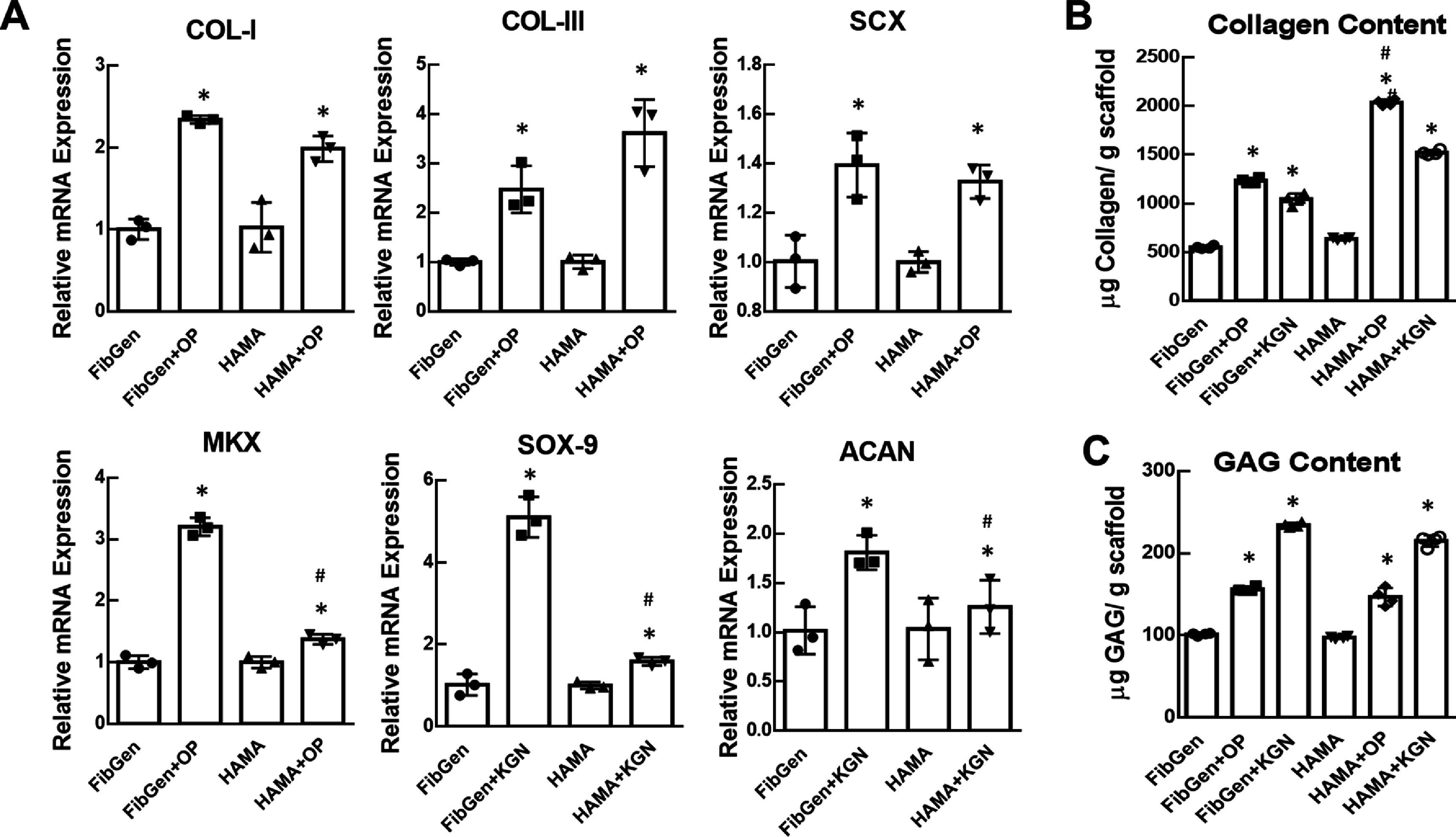
mRNA expressions of differentiation markers (A), and total collagen (B) and GAG contents (C) after 4 weeks culture with MSCs. Fibrogenic differentiation markers, including COL-I, COL-III, SCX, and MKX were significantly increased with OP delivery, and chondrogenic markers, such as SCX-9 and ACAN, were significantly increased with KGN delivery (A). Total collagen was significantly higher with OP and KGN delivery than hydrogel alone control (B). Similarly, GAG contents were significantly higher with OP and KGN delivery (C) (*n* = 3 biological replicates; *:*p* < 0.001 compared to hydrogel alone control; #:*p* < 0.001 compared to FibGen + OP).

qRT-PCR analysis from the *in vitro* samples harvested 4 weeks showed that mRNA expressions of fibrogenic markers, including COL-I, COL-III, SCX, MKX, were significantly increased in scaffolds with Oxo-M and 4-PPBP delivery via FibGen and HAMA as compared to controls without SMs (figure 6(A)) (*n* = 3 biological replicates; *p* < 0.001). Similarly, chondrogenic marker mRNA expressions, such as SOX-9 and ACAN, were significantly increased in the scaffolds with KGN via FibGen and HAMA (figure 6(A)) (*n* = 3 biological replicates; *p* < 0.001). Collagen assays showed significantly higher contents of total collagen in scaffolds delivered with Oxo-M and 4-PPBP or KGN as compared to control without SMs (figure 6(B)) (*n* = 5 per group; *p* < 0.001). GAG assays showed significantly higher contents of total GAGs in the scaffolds with KGN, with a notable increase in scaffolds with Oxo-M and 4-PPBP (figure 6(C)) (*n* = 5 per group; *p* < 0.001).

### 3.5. In vivo formation of multiphase fibrocartilaginous tissue matrix

**Figure 7.**
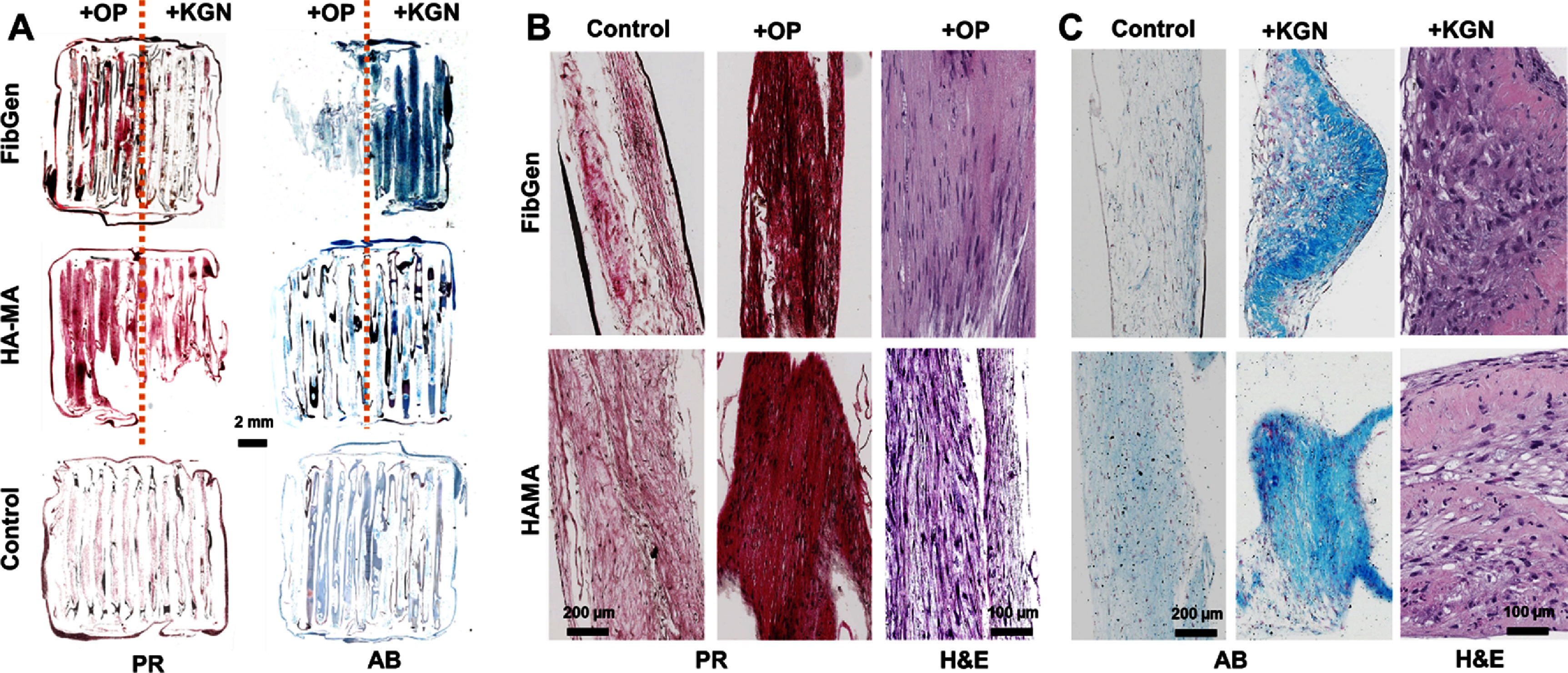
Multiphase fibrocartilaginous tissue formation *in vivo* by 4 weeks. Slide scanned sections show distinct distribution of fibrous and cartilaginous matrix (A). OP-delivered region exhibited densely aligned fibrous tissues with spindle-shaped fibroblast-like cells (B). The KGN-delivered region showed a cartilaginous matrix populated with rounded chondrocyte-like cells (C).

The *in vivo* tissue constructs harvested at 4 weeks revealed a clear distinction between fibrous tissue and cartilaginous tissue formed at region with Oxo-M + 4-PPBP and KGN, respectively (figure 7(A)). PR staining showed dense collagenous matrix formed at the region with Oxo-M + 4-PPBP, in comparison with control (figure 7(B)). High magnification H&E sections showed spindle-shaped fibroblast-like cells were dominantly populated in the region delivered with Oxo-M and 4-PPBP (figure 7(B)). Similarly, the chondrogenic region delivered with KGN via FibGen and HAMA showed AB-positive cartilaginous matrix in comparison to control without SMs (figure 7(C)). The scaffold region with KGN was also dominantly populated with rounded chondrocyte-like cells (figure 7).

## 4. Discussion

This study provides a comparative data set for the printability of micro-thin hydrogel carriers as integrated into the 3D printing of PCL scaffolds. We have successfully established optimal printing and design parameters to enable spatiotemporal delivery of multiple SMs as incorporated in 3D printing process. The selected hydrogels formed a homogenous, micro-thin coating on each PCL filament, which neither interfered with the process of constructing 3D scaffolds nor altered overall structural properties. As the micro-thick coating is formed along with each 3D-printed PCL fiber, our approach enables spatiotemporal delivery of SMs, maintaining any specific micro-pattern designed in the 3D-printed scaffolds. On the other hand, particular micro-patterns designed in 3D-printed scaffolds do not interfere with SMs delivery efficacy. The delivered SMs via micro-coating of hydrogel were effective in stimulating MSCs toward differentiation into desired tissue types in a spatially controlled manner in the 3D-printed scaffolds. As the hydrogel carriers tested in this study, MDP, HAMA, and FibGen, have shown their potential as effective control-delivery vehicles not only for SMs but also for GFs, cells, and gene therapies [23, 30, 32, 36], the present data with comprehensive printability data may have a wide range of applications for spatiotemporal delivery through 3D-printed scaffolds. In addition, various functionalization and chemical modifications are readily available for the selected hydrogel-based carriers, suitable for specifically targeted delivery [37].

Our novel micro-coating approach significantly contributes to the field by overcoming multiple outstanding limitations of the existing advanced 3D printing technologies. Core–shell bioprinting is one of the promising tools for spatiotemporal delivery combined in a 3D scaffold [38]. However, core–shell bioprinting is limited in the material selection, as soft hydrogel-based materials can be mostly used for core and shell materials, making it suitable for bioprinting cells in the core and GFs in the shell. However, core–shell is mostly not feasible to construct a mechanically demanding 3D-printed structure [38, 39]. In contrast, our micro-coating approach allows the construction of a mechanically robust structural 3D scaffold incorporated with micro-precise spatiotemporal delivery. Dip coating was also used to form spatially controlled delivery of bioactive factors in the 3D-printed solid scaffolds [7]. However, it is limited in the resolution of spatial control as the coating is made by dipping entire scaffolds into hydrogel solution in a way to load factors [7]. More importantly, dip coating cannot provide z-layer-specific coating, limiting the scope of its applications [7]. Our micro-coating approach enables any custom-designed coating patterns throughout the 3D-printed scaffolds, supporting the novelty and significance of our findings.

For controlled delivery of SMs or GFs, the soluble factors can be loaded with these hydrogel carriers, followed by cross-linking, which leads to sustained release primarily controlled by slow degradation of the materials [37]. As a unique self-assembling hydrogel, MDPs are instantly organized into *β*-sheets upon extrusion through a dispensing nozzle that encapsulates SMs or GFs as entrapped in nanofibrous meshes [30], thus resulting in prolonged release [8, 30–32]. Interestingly, despite its proven efficacy in providing sustained release of SMs as an injectable hydrogel [32], MDP in the form of micro-coating showed a relatively lower efficiency in the initial loading and prolonged release of Oxo-M and 4-PPBP compared with HAMA and FibGen. This is likely associated with potential chemical interactions between MDP and PCL or shear thinning during dispensing through a micro-sized nozzle. Although MDP has shown rapid self-healing after experiencing 3D printing-derived shear thinning [8], its capacity to entrap bioactive cues after shear thinning has not been documented to date.

Despite the promising outcomes in spatiotemporal delivery of SMs integrated in 3D printing, our study has several limitations. First, the hydrogel micro-coating may interfere with surface treatments applied on 3D-printed structural filaments. For example, NaOH treatment can be applied to increase the surface roughness of 3D-printed scaffold that leads to accelerated degradation [3, 18]. The effect of such surface treatments has not been tested when hydrogel micro-coating is applied. Second, given the nature of the micro-thin coating, the total amount of loaded SMs can be limited compared to bulk hydrogel carriers. Although our data showed that the bioactivities of released SMs were sufficient to induce MSC differentiation and multiphase tissue formation, future studies are warranted to quantify the maximum loading capacity and duration of release. Third, our *in vivo* study was limited to ectopic tissue formation rather than *in situ* implantation for mechanically demanding tissue targets. Fourth, our *in vitro* release kinetics were tested in PBS, which is likely varied from those under *in vivo* biochemical and physiological environments. However, we have no available technology to trace the *in vivo* release of SMs. Lastly, the presented printing technology requires a unique functionality of 3D bioprinters equipped with multi-head printing and a customizable precise positioning system, which may not be available in certain bioprinter systems.

In conclusion, the spatially controlled, micro-thin coating of hydrogel layers may serve as an efficient to enable micro-precise spatiotemporal delivery of bioactive cues through 3D printed scaffolds. We expect this bioprinting method will be highly beneficial to develop bioactive scaffolds to guide the regeneration of complex inhomogeneous musculoskeletal and craniofacial tissues, including, but not limited to, knee meniscus, tendon enthesis, synovial joints, and temporomandibular joint disc. Our micro-coating approach can form spatiotemporal delivery of profibrogenic and chodrogenic cues in mimicking the regionally variant fibrocartilaginous matrix presented in those complex tissues, as incorporated in 3D-printed anatomically correct scaffolds to replace the whole or parts of the tissues.

## Data Availability

All data that support the findings of this study are included within the article (and any supplementary files).
